# Construction Notes

**Published:** 1896-07-25

**Authors:** 


### CONSTRUCTION NOTES.

### New Northern Hospital, Liverpool.

The site on which the new hospital is to be built covers about 11,000 yards, and is situated around the building which, with its 157 beds, has for so long fulfilled a most commendable and important work in the city. The new hospital is designed for 200 beds, with the large longitudinal pavilions of three storeys in height at right angles to Great Howard Street, the administration block being placed in a central position between them. At the corner of Great Howard Street and Leeds Street is placed the nurses' home for sixty nurses, and all are connected by a main corridor. At the north-east corner of the land the operating and lecture theatres and the out patient department are situated, with a separate entrance and exit, and at the back are placed the laundries, boiler-house, disinfecting house, &c., with the mortuary and post-mortem room in a separate block. The principal entrance to the hospital is in the administration block, which will be approached by a circular drive, and will contain a handsome entrance hall and accommodation for the officials and executive of the hospital. For the general working of the hospital, an entrance has been provided at the end of the main corridor fronting Leeds Street, where there is a large receiving hall, with waiting and casualty receiving rooms and porter's room adjoining. Above the Leeds Street entrance are two small male and female accident wards, easily accessible by two staircases and lifts placed at each end of the main corridor. All the wards are isolated from the main corridor, and each ward has attached to it nurses', kitchen and small dining room for convalescent patients, a pay ward, an isolation ward (each for a single bed), and the usual accessories. There are also three special wards fitted up with tanks for typhoid cases, and two small erysipelas wards. A space under the nurses' home is being provided to be fitted up as a chapel at a future date. The building will be fireproof, and the heating, ventilating, and sanitary arrangements will be on the latest and most approved principles. The disposition of the various parts of the new building is such that, with the exception of a small part of the administrative block, the whole of the new hospital can be erected without interfering with the present existing hopital. The building is, as we have previously stated in these columns, the outcome of a munificent bequest of £250,000 left by Mr. David Lewis to the municipality of Liverpool, £60,000 of which money was devoted to the present purpose of hospital construction.

